# Yearly variation coupled with social interactions shape the skin microbiome in free-ranging rhesus macaques

**DOI:** 10.1128/spectrum.02974-23

**Published:** 2023-09-26

**Authors:** Christina E. Roche, Michael J. Montague, JiCi Wang, Allison N. Dickey, Angelina Ruiz-Lambides, Lauren J. N. Brent, Michael L. Platt, Julie E. Horvath

**Affiliations:** 1 North Carolina Museum of Natural Sciences, Raleigh, North Carolina, USA; 2 Department of Neuroscience, University of Pennsylvania, Philadelphia, Pennsylvania, USA; 3 Bioinformatics Research Center, North Carolina State University, Raleigh, North Carolina, USA; 4 Caribbean Primate Research Center, University of Puerto Rico, San Juan, Puerto Rico, USA; 5 Centre for Research in Animal Behaviour, University of Exeter, Exeter, United Kingdom; 6 Marketing Department, University of Pennsylvania, Philadelphia, Pennsylvania, USA; 7 Department of Psychology, University of Pennsylvania, Philadelphia, Pennsylvania, USA; 8 Department of Biological and Biomedical Sciences, North Carolina Central University, Durham, North Carolina, USA; 9 Department of Evolutionary Anthropology, Duke University, Durham, North Carolina, USA; 10 Department of Biological Sciences, North Carolina State University, Raleigh, North Carolina, USA; 11 Renaissance Computing Institute, University of North Carolina at Chapel Hill, Chapel Hill, North Carolina, USA; Lerner Research Institute, Cleveland, Ohio, USA

**Keywords:** skin microbiome, rhesus macaques, social behavior, year-to-year environment, Cayo Santiago, primates, microbial diversity, 16S rRNA

## Abstract

While skin microbes are known to mediate human health and disease, there has been minimal research on the interactions between skin microbiota, social behavior, and year-to-year effects in non-human primates—important animal models for translational biomedical research. To examine these relationships, we analyzed skin microbes from 78 rhesus macaques living on Cayo Santiago Island, Puerto Rico. We considered age, sex, and social group membership, and characterized social behavior by assessing dominance rank and patterns of grooming as compared to nonsocial behaviors. To measure the effects of a shifting environment, we sampled skin microbiota (based on sequence analysis of the 16S rRNA V4 region) and assessed weather across sampling periods between 2013 and 2015. We hypothesized that, first, monkeys with similar social behavior and/or in the same social group would possess similar skin microbial composition due, in part, to physical contact, and, second, microbial diversity would differ across sampling periods. We found significant phylum-level differences between social groups in the core microbiome as well as an association between total grooming rates and alpha diversity in the complete microbiome, but no association between microbial diversity and measures of rank or other nonsocial behaviors. We also identified alpha and beta diversity differences in microbiota and differential taxa abundance across two sampling periods. Our findings indicate that social dynamics interact with yearly environmental changes to shape the skin microbiota in rhesus macaques, with potential implications for understanding the factors affecting the microbiome in humans, which share many biological and social characteristics with these animals.

**IMPORTANCE:**

Primate studies are valuable for translational and evolutionary insights into the human microbiome. The majority of primate microbiome studies focus on the gut, so less is known about the factors impacting the microbes on skin and how their links affect health and behavior. Here, we probe the impact of social interactions and the yearly environmental changes on food-provisioned, free-ranging monkeys living on a small island. We expected animals that lived together and groomed each other would have more similar microbes on their skin, but surprisingly found that the external environment was a stronger influence on skin microbiome composition. These findings have implications for our understanding of the human skin microbiome, including potential manipulations to improve health and treat disease.

## INTRODUCTION

The skin is the largest organ of the mammalian body and the main barrier against the external world (1). Microbes on the surface of the skin often participate in a symbiotic relationship with host skin cells by playing a significant role in mediating body odor and health (2, 3). Specifically, the immune system of the skin is heavily reliant on the microbiota for defense and tissue repair (4). Certain skin microorganisms, like *Staphylococcus epidermidis*, can directly modulate the expression of inflammatory receptors within the innate human immune system that are necessary for maintaining tissue integrity (5). Furthermore, the skin microbiome influences the length and quality of wound healing via activity of *Pseudomonas* spp. (6). Research comparing human and non-human primate skin microbiota shows that host-microbe co-evolutionary history can be influenced by human hygiene behavior (7). Thus, developing greater insight into factors associated with skin microbial diversity and composition can not only inform future medical interventions, but also bridge unknown gaps in the evolutionary history of the human microbiome. Studying how microbiomes on non-human primate skin vary with behavior and health may thus offer new evolutionary and translational insights for humans.

Recent metagenomic studies identify interactions between gut microbiota and social behavior in non-human animals (8 – 12). Among Welsh Mountain ponies, more frequent social interactions were associated with increased similarity in gut microbiome composition (13). Gut microbiota of wild baboons and chimpanzees were more homogenous in individuals that socialized more frequently, compared with individuals who shared the same diet or ancestral lineage (10, 11). Baboon males disperse from their natal group and the duration of male residency has been correlated with higher microbiome similarity (14). In colobus monkeys, gut microbial beta diversity was specific to individual social groups, consistent with local transfer of microbes either through direct interaction or shared spaces (15). Similarly, humans who interacted regularly within the same household, including with spouses and pets, possessed similar gut microbiome composition (12).

Several studies show that the environment also influences the composition of the gut microbiome. Human gut microbial composition varied with ambient environmental ozone and concentrations of nitrogen oxide and nitrogen dioxide (16). Tanzanian chimpanzees shared more similarity in gut microbiome composition during wet seasons when they were more sociable, and less similarity over periods of fewer social interactions during the dry seasons (17). In monkeys, the diversity of the gut microbiome can be explained by a combination of social behavior and seasonal change, or seasonal changes alone (18 – 21). Taken together, it is clear that social behaviors and environmental factors interact to shape the composition and diversity of the gut microbiome.

Several studies of the skin microbiome in mammals show associations between microbial diversity and seasonality, temperature, humidity, or geographic location (22, 23). For instance, in fruit bats, seasonality was the largest driver of skin microbial diversity (22). Several human microbiome studies show that temperature and humidity changes lead to shifts in skin microbial taxa abundances [reviewed in reference (24)]. In voles, alpha and beta diversity of the skin microbiome clustered according to geographic area, whereas the gut microbiome in the same animals did not, suggesting that the skin microbiome is more sensitive to the environment (25). In 10 non-human mammalian orders, the most significant factor influencing skin microbiota after accounting for host taxonomic order was geographic location (26).

Despite these advances and their translational and evolutionary importance, the relationships between the skin microbiome, age, sex, social factors (such as group membership and behavior), and the environment in non-human primates remain unclear. We address this gap by investigating the bacterial diversity and composition of the skin microbiome in the free-ranging colony of rhesus macaques on Cayo Santiago Island, a unique and well-established living laboratory for comparative and translational research. We hypothesize that, first, monkeys who interact more with other monkeys or who have more similar social behaviors will possess more similar skin microbial composition and diversity. Second, we hypothesize that skin microbial diversity will vary across sampling periods due to naturally occurring changes in the environment (such as temperature, rainfall, humidity) from 1 year to the next.

## MATERIALS AND METHODS

### Study subjects and behavioral data collection

Animal subjects (*n* = 93) were members of five different social groups and ranged in age from 4 to 27 years old among a free-ranging population of 1,600 rhesus macaques residing on Cayo Santiago Island, Puerto Rico. The founding population of the research colony included 409 rhesus macaques moved from India to Cayo Santiago in 1938 (27). Colony members self-organize into social groups, demonstrate various forms of social behaviors, most notably grooming (28, 29), and reproduce in a semi-natural environment with minimal external manipulations. A provisioned diet consists of commercially available monkey chow and water, supplemented by natural vegetation. Throughout the year, males tend to transfer to non-natal social groups after reaching reproductive age (3–4 years), whereas females remain in their natal group (28, 30).

Biological samples were collected during sampling periods. These occurred approximately 2 months after each birth season, which vary from year to year. The sampling periods for this project spanned between 2 months (October–December) to 4 months (October–February). Apart from yearly trap-and-release activities for biological sampling, as well as intermittent population control over the past several decades, there are minimal human interventions (31). These procedures are covered under the IACUC protocol #A6850108 of the Medical Sciences Campus, University of Puerto Rico.

All rhesus macaques in this study were individually identified, recognized, and monitored by observers and census takers. Extensive behavioral data, which included 394 hours of observations (mean of 4.24 hours per animal), were collected for a subset of animals to calculate metrics of dominance rank, grooming, and nonsocial, or self-directed, behaviors (SDBs: self-grooming and scratching). We operationalized behavioral data in several ways (Table 1): social group membership (five groups: F, HH, KK, R, and V), ordinal dominance rank, and rates of grooming received, grooming given, total grooming (adding received and given rates), and self-directed behaviors. The majority of animals both received and gave grooming; a smaller subset of animals only received or only gave grooming, but not both. For behavioral analyses, we included animals that were observed for more than 2 total hours of individual 10-minute focal samples (>12 observation samples). Dominance was determined for members of each sex separately through both direction and outcome of win/loss interactions within social groups, and monkeys were accordingly assigned ordinal ranks, with lower numbers representing higher dominance ranks. Per animal rates of grooming and self-directed behaviors were calculated by dividing the number of minutes of each behavior by the total number of minutes observed within the 8–10 months of observation prior to the collection of the skin swab. Grooming was defined as running the hands or mouth through the hair of another monkey. Grooming bouts were recorded if grooming duration exceeded 5 seconds. A new bout of grooming was recorded if the identity of more than one partner changed or if there was a pause lasting longer than 15 seconds (32). Self-grooming was defined as running the hands or mouth through one’s own hair and recorded if duration exceeded 5 seconds. A new bout of self-grooming was recorded if 15 seconds lapsed in which no self-grooming occurred. Scratching was defined as rapid and repeated movement of the hand or foot across the skin. A new scratch was recorded each time the hand or foot was removed from contact with the area of the body being scratched and returned to a passive position (e.g., arm returned to the side of their body) or to the original position of the hand or foot prior to the scratch. A new scratch was also recorded when the area of the body being scratched was changed (e.g., two scratching events were recorded if an individual moved their nails across a section of their arm, followed by a section of their head).

**TABLE 1:** Skin swab samples by category and sampling period^*a*^

		Social group					Behavioral	Sampling period		
Sub-category	All	F	HH	KK	R	V	Dominance, grooming interactions, self-grooming, self-scratching	2013	2014	2015
Number of samples	78	43	4	8	11	12	53	42	3	33
Females	45	30	2	3	6	4	38	26	1	18
Males	33	13	2	5	5	8	15	16	2	15

^*a*^ Sampling period 2014 samples removed for sampling period statistical analyses only.

### Sample collection, DNA extraction, and sequencing

A subset of the research colony was trapped and released annually for biological sampling (sampling period) as part of multiple collaborative research projects exploring behavior, ecology, demography, life history, and genetics (28, 29, 31 – 33). Skin microbes were collected from both the left and right axillae of sampled macaques. Axillary areas are a more protected region of the skin microbiota, given their relative lower exposure from soil and other environmental factors. Microbiome swabs were stored at −80°C at the research field station, shipped on dry ice to North Carolina, and stored again at −80°C until extraction and sequencing.

For this study, we selected 93 left axilla samples for microbial DNA isolation and next-generation sequencing of the 16S V4 rRNA region from three different rhesus macaque sampling periods (2013–2015) and five different social groups. Each animal was represented by only one replicate swab in the data set. Environmental changes in this study corresponded to natural differences (e.g., humidity, temperature, etc.) that occurred from one sampling period to the next, measured across each sampling period spanning the years 2013 to 2015. The 2013 sampling period occurred from October 2013 through February 2014; the 2014 sampling period occurred from October 2014 through December 2014; and the 2015 sampling period occurred from October 2015 through December 2015. The month and year of sample collection was recorded and subsequently grouped according to sample period. For data analyses, all microbiome sequencing reads were rarefied (see below), and as a result, our assessments of social group and seasonality encompassed 78 monkeys (84% of the 93 monkeys with skin microbe sequence data were retained after rarefaction), and for some analyses, the 2014 sampling period was removed due to unbalanced sampling. Our analyses of behavioral measures (dominance, grooming, SDBs) included a subset of 53 monkeys (57% of the total sequenced).

Each axilla was swabbed using dual-tipped rayon swabs (BD BBL culture swabs B4320135). One swab tip from each sample was used for microbial DNA extractions while the other tip remained stored at −80°C as a technical replicate. Extractions were carried out using PowerSoil DNA Isolation kits (MOBIO Laboratories, CA, USA) according to the manufacturer’s protocol with the following modifications. First, the swab tip was cut with sterilized scissors and vortexed in the bead lysis tube. Second, for the final elution step, sample columns were warmed to 55°C with 60 µL of elution buffer C6. Each kit contained a kit control, which was an un-sampled sterile rayon swab that was processed with the other samples. Isolated DNA was stored at −20°C until DNA amplification with standard polymerase chain reaction (PCR) methods and 16S rRNA primers “515F” and “806Rmod” (Table S1). DNA amplification reactions were prepared within a PCR workstation laminar flow hood to reduce contamination. 5 PRIME HotMasterMix (QuantaBio, CA, USA) was mixed with 5 µL of DNA template and 0.5 µL (10 µM) primers in 25 µL reactions and amplified on an Eppendorf thermal cycler (Eppendorf, Germany) using the following program: 94°C for 3 minutes; 35 cycles of 94°C for 45 seconds, 50°C for 60 seconds, and 72°C for 90 seconds; followed by 72°C for 10 minutes and held at 4°C.

Reactions were checked for the expected 292 base pair (bp) fragment using gel electrophoresis. PCR products were cleaned individually using Zymo DNA Clean and Concentrator kit (Cat# D4014) per the manufacturer’s protocol and eluted in 27 µL of elution buffer. Each product was individually ligated with unique adapter-indexed fragments (Table S1) using the KAPA Biosystems Hyper Prep kit (Roche Sequencing & Life Science, Pleasanton, CA) to create individual sequencing libraries. The first (“1st”) set of microbiome samples (28 out of 78) (Table S2) was ligated using half-volume reactions: 25 µL clean DNA and 5 µL of end-repair and A-tail master mix, held in a thermocycler at 20°C for 30 minutes, and 65°C for 30 minutes; then adapter ligation reaction master mix was added to make a 55 µL reaction, held on the thermocycler at 20°C for 15 minutes, then 4°C for 5 minutes. The second (“2nd”) set of samples (50 out of 78) (Table S2) was ligated with indexed adapters using full size reactions: 60 µL for end-repair and A-tailing and 110 µL for ligation reactions; then the remainder of steps followed as with half-volume reactions. All ligation products were cleaned using Ampure beads (Agencourt Ampure XP PN A63880). Qubit dsDNA HS Assay Kit (Invitrogen, OR, USA) was used to quantify all final individual products and an equal mass of each was pooled together in one tube for each library. The first set of samples created one library pool, which was quantified and verified for 450 bp size using Agilent 2200 TapeStation Analyzer (Agilent, CA, USA). The second set of samples was pooled, followed by quantification and fragment size verification using a high sensitivity DNA Agilent 2100 BioAnalyzer (Agilent, CA, USA). Both library pools were sequenced on the Illumina MiSeq next-generation sequencing instrument (Illumina, CA, USA) at the North Carolina Museum of Natural Sciences Genomics and Microbiology Research Laboratory using the Illumina MiSeq V3 600 cycle sequencing reaction kit in two different sequencing runs. The first sequencing run was loaded at 12pM and 25% PhiX (437 k/mm^2^ cluster density). The second library was loaded at 16pM and 20% PhiX (1,100 k/mm^2^ cluster density).

### Amplicon sequence processing

Sequenced reads were processed using the QIIME 2 pipeline (v2019.10) and its plugins or algorithms (q2) (34). The raw reads were demultiplexed and filtered for quality using the q2 demux plugin. We generated amplicon sequence variant (ASV) tables for all sequences in each of the two libraries using DADA2 (v 1.10.1), as implemented using the q2 dada2 denoise-paired command (35). Read processing steps included read quality filtering, read trimming and primer removal (truncated forward reads at 250 bp, trimmed left 19 bp; truncated reverse reads at 220 bp, trimmed left 20 bp), merging denoised paired-end reads, and filtering the merged reads for chimeras. For each of the two ASV tables, the Decontam R package (prevalence method) was used to identify contaminant ASVs using their respective kit control (36). The Decontam R Prevalence method identified 13 out of 18,935 ASVs as contaminants in the set of samples using the DNA isolation kit “C2,” which had a frequency range of 0.000026 in two samples to 0.000101 in eight samples and a prevalence range of 0.025 in two samples to 0.1 in eight samples. The same method identified 17 out of 34,985 ASVs as contaminants in the set of samples using the DNA isolation kit “Cs,” which had a frequency range of 0.000003 in 2 samples to 0.000303 in 13 samples and a prevalence range of 0.01 in 2 samples to 0.09 in 13 samples. Due to the extremely low frequency of these removed taxa, their removal should not impact how these were distributed in our data set across sequencing libraries or other variables. The ASV tables were filtered to remove contaminate ASVs, imported back into Qiime2 artifact format, and then combined into one ASV table using q2-feature-table merge. By running the q2-feature-table filter-features plugin, the merged table was filtered to remove ASVs that had fewer than 10 reads total. The resulting file was an ASV table (24,464 ASVs) of features for the 93 samples and the accompanying representative sequence file was also filtered to only include these features.

The distribution of ASV sequence lengths was inspected in R (v 3.6.1) using DADA2 (v 1.12.1) (35). A total of 3,152 ASVs (12.9%) deviated from the expected amplicon length (~254 bp), and were removed as potential non-target hits. The 21,312 ASVs whose sequence lengths were in the range of 252–255 bp were saved for downstream processing. After examining the ASV table, 15 of the 93 samples were removed based on previous analysis (see rarefaction below) due to low sample sequencing depth and 78 samples were retained out of the total sequenced 93 samples (20,905 ASVs remained). ASVs were clustered in Qiime2 at 100% sequence identity using the open reference method (q2-vsearch) (37) against the Silva nr 99 reference database, v132 (16S only), to cluster those ASVs that existed on opposite strands and only differed in base pair at their sequence start. Taxa were assigned to the resulting 17,988 ASVs using the assignTaxonomy and addSpecies functions (with tryRC = TRUE) in R using DADA2 with the Silva reference database (v132) (38). The data processing steps and resulting ASV counts are listed in (Table S1). The ASV table with all samples was adjusted for batch correction for the two different sequencing libraries using MMUPHIN in R (39) and the metadata column “SeqLib” with co-variants “KitCntrl” and “SamplingPeriod.” Each co-variant has a number of samples that overlap with one or the other SeqLib sets. Batch correction with these co-variants was chosen to give more power to the overall data set for which some social groups have small sample numbers. Despite controlling for the sampling period variable with MMUPHIN, biological differences between the samples collected from the two sampling periods are still observed. This allowed us one standard batch-adjusted ASV table to use in analyses for both hypotheses.

### Data analyses

#### Feature rarefaction and rooted tree generation

A rooted phylogenetic tree was generated on the 93 samples from a multiple sequence alignment of representative sequences using the q2 algorithm align-to-tree-mafft-fasttree (40 – 42). Next, the q2-diversity core-metrics-phylogenetic plugin was used to rarefy to a sequencing depth of 35,000, based on the q2 alpha rarefaction curves for Faith’s phylogenetic diversity (Faith’s PD), via q2-rarefaction (43 – 45). The chosen sequencing depth allowed the inclusion of the most phylogenetically diverse set of microbiota from our data set giving a comprehensive microbiome while keeping a majority of samples. Choosing a lower sequencing depth would exclude valuable microbiota. At 35,000 sampling depth, 17,812 ASVs and 78 samples remained in the rarefied ASV feature table. A rooted phylogenetic tree was re-generated on the 78 samples from a multiple sequence alignment of clustered representative sequences using the q2 align-to-tree-mafft-fasttree. The rooted tree, the resulting metadata file (Table S2), and rarefied ASV feature table (Table S3) were used for downstream analysis where required. We characterized the rarefied ASV feature table as the “complete” microbiome table. For sampling period analysis only, this table was further filtered to remove three 2014 sampling period samples, retaining only 75 samples and 17,498 ASVs from sampling periods 2013 and 2015.

#### Core and non-core microbiome features

The complete microbiome table containing the rarefied ASVs was collapsed to the taxonomic level of phylum and core features identified for visualization, via qiime2-2021.04 q2-core features (46). The core features 1.0 list (e.g., features present in 100% of samples) was used to filter the complete microbiome table and output a “core” microbiome feature table, via q2-filter-features. The same core features were then removed from the complete microbiome table to output a “non-core” microbiome feature table. Core and non-core feature tables were then exported to biom format (47). For sampling period analysis only, phyla core and non-core tables were filtered to retain only 75 samples from 2013 and 2015 sampling periods.

The core microbiome was calculated for the family level of classification at 1.0 (e.g., all animals have the core bacteria; Tables S4 and S5) using the program Calypso (48). Given the large diversity of bacteria in our data set, the family-level classification was used to more easily resolve the core features. Calypso was used to generate the Venn diagram output and a detailed table of abundances for taxa in each sub-group. Sampling period 2014 was included here as these tables were shown only for the distribution of taxa and not for statistical analysis.

#### Relative abundance

Relative abundances from the rarefied table were calculated as an average for each taxon across all samples within a specified variable and are presented for complete, core, and non-core microbiomes. The three different feature tables (complete, core, and non-core) were individually merged with the taxonomy table and animal metadata in R. For the 78 samples assigned to a social group, the rarefied ASV table included 17,812 ASVs. The ASVs were agglomerated at the phylum level using the R package phyloseq (v 1.36.0) (49), which resulted in 51 phyla. For the 75 samples assigned to the 2013 or 2015 sampling periods, the rarefied ASV table included 17,498 ASVs. When these ASVs were agglomerated at the phylum level, there were again 51 phyla. In both cases, four of the phyla did not have an assigned taxa. Bar plots displaying relative abundance of phyla across (i) social groups and (ii) sampling periods were created in R (v 4.1.0) using the ggplot2 package (50, 51). To identify potential significant differences between phyla across different social groups and across different sampling periods, we used Kruskal-Wallis (KW) (52) or Wilcoxon signed rank (53) tests in the R package ggpubr (v 0.4.0).

The abundances were also assessed using a compositional approach (54). With this method, one examines the ratios between taxa such that a change in taxa abundance is relative to the other taxa. An application of this approach can be seen in a study of the vaginal microbiome in expectant Brazilian mothers (55). The zCompositions R package (v 1.4.0-1) was used to replace zero values with non-zero values using the count zero multiplicative method (56). The relative abundances for each sample were calculated and then center log ratio (CLR) transformed using the R package CoDaSeq (v 0.99.6) (57). Boxplots were generated using ggpubr (v 0.4.0) for each of the phyla with an assigned taxa, with CLR-transformed relative abundance values displayed on the y-axes. For comparisons across social groups, an overall *P*-value is shown on each plot and was calculated using a Kruskal-Wallis test. Pairwise comparisons between social groups were calculated using Wilcoxon tests. For sampling period, differences between the 2013 and 2015 seasons were calculated using Wilcoxon tests.

#### Alpha diversity analysis

We calculated indexes of Faith’s PD for the 78 rarefied samples (q2-diversity) using the rarefied feature table at 35,000 sampling depth (44). The Faith index values displayed a normal distribution following a Shapiro-Wilk test of normality (*P* = 0.786) (58). After establishing normalcy, Faith’s PD values were compared against the categorical variables of social group and sampling period (44, 59). Tukey’s honest significant test was performed on analysis of variance (ANOVA) to examine pairwise comparison between groups and to correct for multiple comparisons (60). A Student’s *t*-test was performed between sampling periods (61). All 78 samples were analyzed by Faith’s PD for social group and 75 samples for sampling period, while the subset of 53 samples was analyzed for behavior, including total grooming, grooming received, grooming given, and SDBs. Analyses were performed in R Studio using the vegan package, with plots visualized using ggplot2 (50, 62). To compare the Faith’s PD index on a continuous variable, a linear plot with fitted regression line was visualized in R using ggplot2 and *P*-values for statistical significance were determined (59).

Likewise, Shannon diversity indexes for the 78 rarefied samples were generated using q2-diversity and the rarefied feature table at 35,000 sampling depth (34, 45). Using the Shapiro-Wilk test of normality, we found Shannon index values in a non-normal distribution for the complete microbiome (*P* = 0.000943) and for the non-core microbiome (*P* = 0.00000365); however, values were normally distributed for the core microbiome (*P* = 0.9722) (58). For the core microbiome, differences in Shannon diversity across different social groups were statistically tested using ANOVA and across sampling periods (75 samples) using a Student’s *t*-test (45, 59). For the complete and non-core microbiomes, significance (52) was determined using a Kruskal-Wallis test, the non-parametric equivalent of ANOVA (52), or for pairwise comparisons, we used the non-parametric Wilcoxon signed rank test (53). All statistical analyses were performed in R Studio using the vegan package, with plots visualized using ggplot2 (50, 60). We tested and visualized Shannon diversity according to age and sex variables for 78 samples, according to sampling period for 75 samples, and according to ordinal dominance ranks and the other behavioral metrics (described above) for 53 samples using linear regression plots in R (63).

#### Beta diversity analysis

Weighted and unweighted UniFrac distance matrices (q2-diversity) were calculated and plotted on the Principal Co-ordinates Analysis (PCoA) axis, with beta group significance determined through the adonis2 function in the R packages phyloseq and vegan (62, 64 – 70). The 78 samples assigned to social groups and the subset of 75 samples assigned to the 2013 and 2015 sampling periods were tested according to their categorical variables, and plots were visualized in RStudio using the package ggplot2 (50).

#### Significantly different taxa

To more fully understand differentially abundant taxa, we compared ASV features between sampling periods (2013 versus 2015), using the program ALDEx2 (q2-aldex2) (71 – 73). These were chosen for comparison because they showed a significant difference in pairwise alpha diversity using Faith’s PD calculations. The subset of samples collected during both sampling periods (*n* = 75) were filtered from the clustered and batch adjusted feature table of non-rarefied counts. Significance was calculated using Welch’s *t*-test and Wilcoxon test on CLR transformed counts. Benjamini–Hochberg (BH) corrected values were calculated for each feature (74). Results using a corrected Welch’s *t*-test were displayed in preliminary volcano and effect plots, and differences were extracted using default parameters (significance threshold = 0.15; effect threshold = 0; difference threshold = 0). Significant differential features and their calculated values were then exported from QIIME2 and imported into R. We represented all pairwise comparative differences between 2013 and 2015 in a single volcano plot created with the R packages tidyverse v1.3.0 (75), ggrepel v0.8.2, and ggplot2 v.3.3.2 (50), with values depicting log2(fold change) plotted against −log10 transformed *P*-values.

#### Environmental weather data

Knowing that weather changes from 1 year to the next can have an impact on environment of microbes, we retrieved local climatological data from a weather station at Roosevelt Roads Naval Station, Ceiba, Puerto Rico using online archives maintained by the National Oceanic and Atmospheric Administration (https://www.ncdc.noaa.gov). The weather station is located approximately 14 km from Cayo Santiago Island. Daily measurements were compared for the entire months of sampling periods in 2013 and 2015. We assessed daily averages for dew point temperature, dry bulb temperature, wet bulb temperature, relative humidity, sea level pressure, station pressure, wind speed, as well as the maximum and minimum dry bulb temperature, departure from normal average temperature, peak wind speed, daily precipitation, and daily sustained wind speed. Weather station data were compiled for 377 different days, including 140 days spanning 1 October 2013 through 25 January 2014 (2013 sampling period; no data available for February 2014); 121 days spanning 1 October 2014 through 30 December 2014 (2014 sampling period); and 116 days spanning 1 October 2015 through 31 December 2015 (2015 sampling period). Missing data were coded as not available, and trace rain measurements (T) were converted to 0.001 inches for statistical purposes (two-sample *t*-tests).

#### Disease annotation of significant taxa

MicroPattern was used to evaluate the list of differentially abundant bacteria according to sampling periods. MicroPattern is an open source web-based tool that uses microbial terms to run an algorithm of enrichment analysis on a database of microbes and associated diseases, phenotypes, or traits and calculates the disease similarity. After submitting the list of our top significant taxa (see results) into the online portal (76), the tool returned statistical output for potentially pathogenic bacteria.

## RESULTS

We identified 17,812 features (ASVs or amplicon sequence variants) of the 16S rRNA V4 region from the rarefied data set of 78 skin axillary swabs from rhesus macaques living on Cayo Santiago in Puerto Rico. An assessment of the overall microbiome composition revealed a total diversity spanning 47 known phyla, plus 160 unknown features (sequences) of bacteria or Archaea. Within this total composition, the most abundant phyla, as measured by relative abundance, comprised *Firmicutes* (52.1%), *Proteobacteria* (18.2%), *Bacteroidetes* (11.5%), and *Actinobacteria* (9.6%), which match the most abundant phyla on human skin, albeit with differing proportions (7, 77). The most abundant phylum on macaque skin, *Firmicutes*, was present in all monkeys and approached the proportions reported in previous studies of rhesus oral microbiome (54.7% relative abundance) (78). Several additional phyla, including *Chloroflexi, Cyanobacteria, Epsilonbacteraeota*, and *Tenericutes*, encompassed 5.1% of microbes on rhesus macaque skin with the remaining other phyla totaling 3.3% in relative abundance. The taxa represented by low abundance create a dramatically more diverse microbiome for rhesus than human skin. Of the identified unique taxa, we found 108 classes, 240 orders, 415 families, 1195 genera, and 515 species in the skin microbiome of the rhesus macaque. The top 30 genera for all animals were identified and listed in Table 2. At the genus level, the two highest relative abundant genera were *Lactobacillus* (7.84%) and *Streptococcus* (4%) in all animals (Table 2). *Staphylococcus*, one of the most commonly found genera on human axillary skin (79), was third most abundant at 2.8%. *Corynebacterium*, another common human skin axillary microbe (79), was found to be only 0.7% in abundance on macaque skin. Other soil-related bacteria such as *Ruminococcus_1* were 1.5% of total genera. *Prevotella_9* makes up 1.3% and four other *Prevotella* genera less than 0.6% each. *Propionibacteriaceae* genera are present at extremely low amounts (less than 0.002% collectively).

**TABLE 2:** Top 30 genera in all 78 macaques and the percent relative abundances of each individual genus out of the total genera abundance

Genus	Percent relative abundance
*Lactobacillus*	7.8%
*Streptococcus*	4.1%
*Staphylococcus*	2.8%
*Lachnospiraceae*_g.	2.8%
*Acinetobacter*	2.7%
*Faecalibacterium*	2.6%
*Ruminococcaceae (UCG-005*)	2.1%
*Ruminococcaceae (UCG-008*)	1.9%
*Sphingomonas*	1.9%
*Chloroplas:*_;g.	1.6%
*Ruminococcus_1*	1.5%
*Kurthia*	1.4%
*Prevotella_9*	1.4%
*Blautia*	1.3%
*Mollicutes (RF39*);_;g.	1.1%
*Rikenellaceae (RC9_gut_group*)	1.1%
*Alloprevotella*	1.1%
*Ruminococcaceae (UCG-002*)	1.0%
*Campylobacter*	1.0%
*Subdoligranulum*	0.9%
*Actinobacillus*	0.9%
*Fusobacterium*	0.9%
*Ruminococcaceae*_g.	0.9%
*Agathobacter*	0.8%
*Glutamicibacter*	0.8%
*Nocardioides*	0.8%
*Ruminococcaceae (UCG-014*)	0.8%
*Corynebacterium*	0.7%
*Prevotellaceae (UCG-003*)	0.7%
*Succinivibrio*	0.6%

### Age and sex effects and alpha diversity

**Fig 1:**
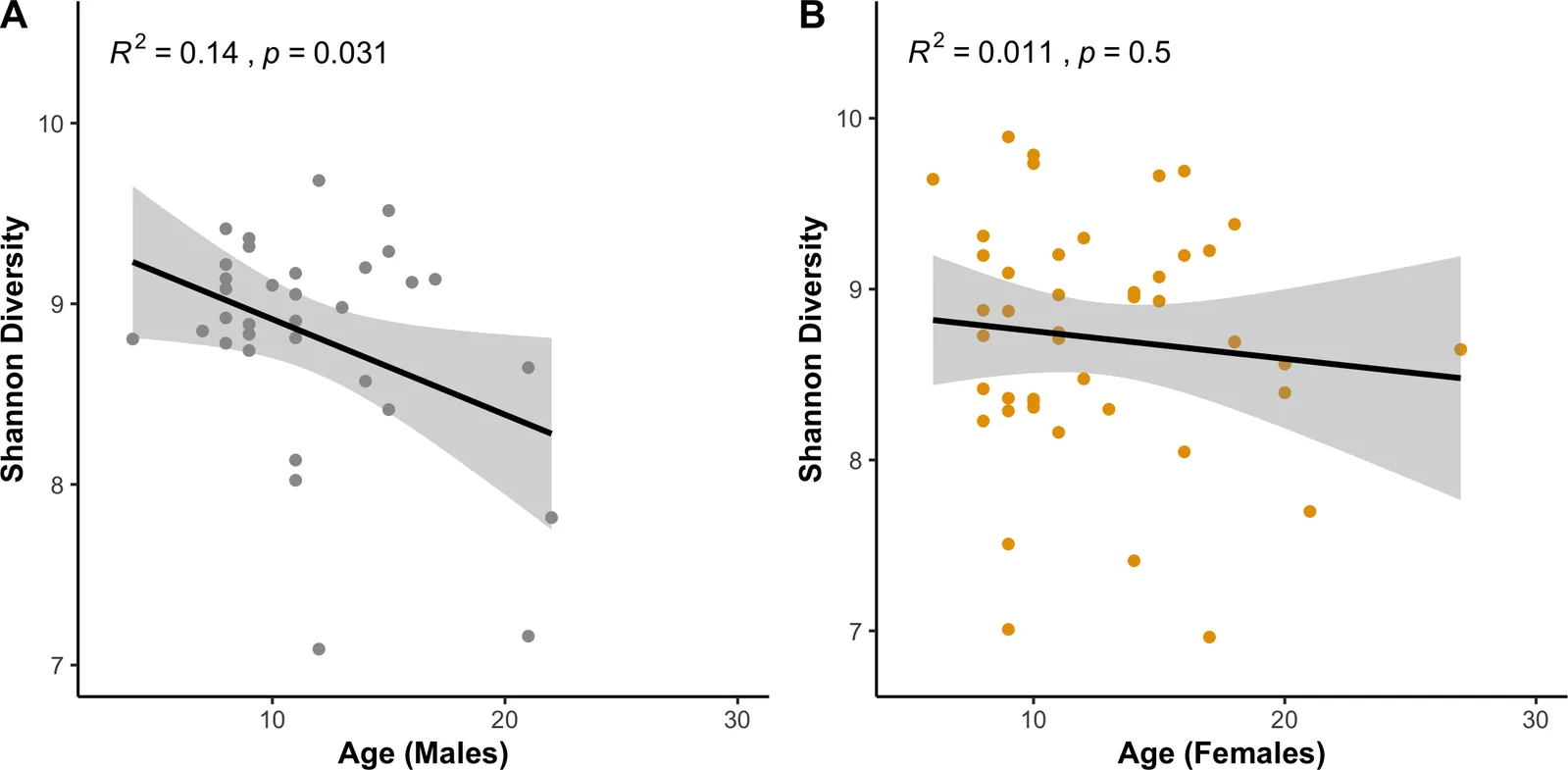
Shannon diversity regression plot as a function of age for males (**A**) and females (**B**).

Human skin microbiome studies have indicated variation based on age and sex (80, 81), so we assessed the impact of these factors in the rhesus macaques. By assessing alpha diversity and richness in all animals according to age and sex (Fig. 1), we found a negative association between age and Shannon index of skin microbiota diversity for males (*R* ^2^ = −0.14, *P* = 0.031) (Fig. 1A), but no directional relationship for females (*P* = 0.5) (Fig. 1B).

### Social group and skin microbiome composition

**Fig 2:**
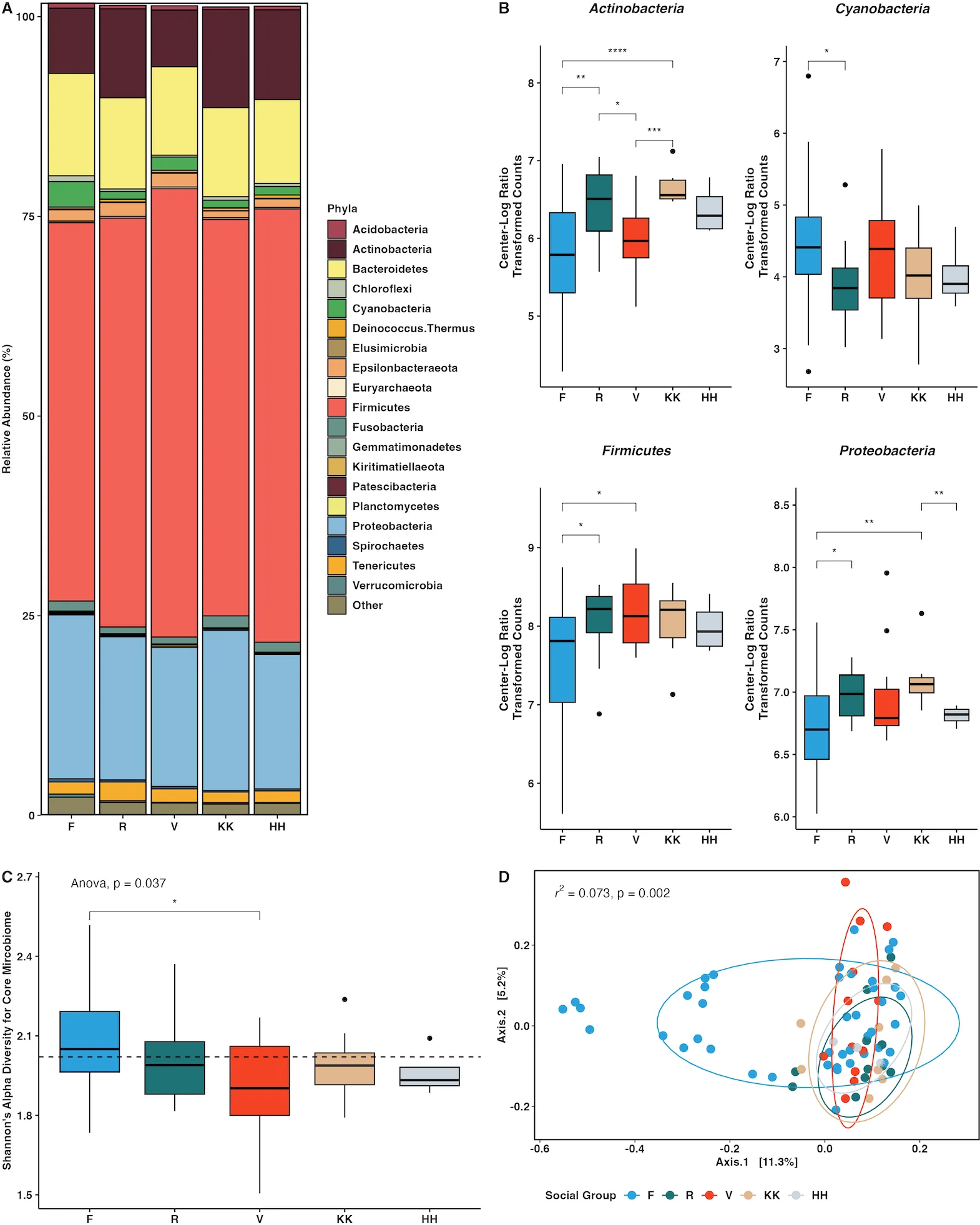
Summary of microbiome diversity for social groups F, HH, KK, R, and V. (**A**) Bar plot displaying the average relative abundances of the top 20 representative phyla per group. (**B**) Box plots representing the taxonomic differences for specific phyla among social groups, with significance assessed using a KW test on CLR transformed relative abundance values: *Actinobacteria* (KW *P* = 0.00013), *Cyanobacteria* (KW *P* = 0.12), *Firmicutes* (KW *P* = 0.044), and *Proteobacteria* (KW *P* = 0.0096). Pairwise comparisons between groups were assessed using *t*-tests; the symbolic number coding of *P*-values is as follows: *, *P* ≤ 0.05; **, *P* ≤ 0.01; ***, *P* ≤ 0.001; ****, *P* ≤ 0.0001. (**C**) Box plots representing alpha diversity using Shannon’s H for the core phyla by social group. Asterisk above social groups F and V indicates significance (*p*.adj = 0.02). (**D**) PCoA plot showing significant association between beta diversity as measured by unweighted UniFrac and social group membership (Adonis2 *R*2 0.073).

We first examined the microbial taxonomy for all 78 animals living across five social groups (F, KK, R, HH, V). We calculated relative abundances (sample averages) of the top 20 phyla per social group using the complete microbiome table of features (Fig. 2A). Patterns of phyla abundance were generally consistent across social groups, although the proportion of each taxon varied slightly according to social group. The most abundant phylum, *Firmicutes*, varied in relative abundance from a minimum of 47% in group F to a maximum of 56% in group V. The next three most abundant phyla included *Proteobacteria* (min: 16.8% in group HH; max: 20.5% in group F), *Bacteroidetes* (min: 10.5% in group HH; max: 12.8% in group F), and *Actinobacteria* (min: 7% in group V; max: 12% in group KK). *Cyanobacteria* comprised 0.97% abundance in group R and 3.2% in group F.

We calculated CLR-transformed relative abundances of each known phyla and identified statistically significant differences by KW test (*P* ≤ 0.05) between pairs of social groups (Fig. 2B). For instance, *Actinobacteria* was less abundant in group F compared to group R (*P* = 0.0019) and also compared to group KK (*P* = 0.000033). *Actinobacteria* was less abundant in group V compared with group R (*P* = 0.032), and compared with group KK (*P* = 0.00071). Members of group R had less *Cyanobacteria* than members of group F (*P* = 0.015). *Firmicutes* was less abundant in group F than in group R (*P* = 0.032) or in group V (*P* = 0.022). *Proteobacteria* was more abundant in group R than in group F (*P* = 0.029), while members of group KK had higher relative abundances of *Proteobacteria* compared with members of group F (*P* = 0.0026) and members of group HH (*P* = 0.0081).

### Social group and total microbiota alpha diversity

Social group was next examined for microbial diversity of the complete microbiome for the five social groups. We assessed our first hypothesis about whether diversity of bacteria would vary by social group since individuals in the same social group were more likely to interact with each other, to the exclusion of members of other social groups. Based on various diversity measures of bacteria using the complete microbiome feature table, we found no significant differences in alpha diversity by social group [Shannon KW *P* = 0.75; Faith’s PD ANOVA Pr(>F) value 0.22] (Fig. S1A and B). Social groups R and V displayed the most pairwise difference in mean for Shannon alpha diversity, while social groups F and V showed the highest pairwise difference in mean Faith’s PD alpha diversity (0.24 and 0.12 adjusted *P*-values, respectively).

### Social group and core microbiome versus non-core microbiome taxonomies and diversity

We parsed core and non-core ASV feature tables to the level of phylum. The core phyla are separate from the least common non-core phyla that were found only in a subset of samples in some groups, as represented in bar plots (Fig. S1C and D). We observed differences in relative abundances between core taxa that are common taxa living on the skin of rhesus macaques, some of which were significant in our assessment of the complete microbiome (Fig. 2B). The proportions show more variability between social groups (e.g., social groups F and R are significantly different in all four taxa) for the core phyla, as described in the complete data set .

Shannon diversity of the core microbiome significantly differed across social groups (ANOVA; *P* = 0.037; Fig. 2C), suggesting that the most consistently present phyla found among all the samples varied in abundance by social group. Specifically, group V displayed less diversity compared to the other groups. The most notable difference in core microbiome alpha diversity occurred between social groups F and V (adjusted *P* = 0.0211, Tukey multiple comparisons of means at a 95% confidence level).

We next examined family-level classifications for the core microbes in order to better understand the diversity between social groups at a finer scale (Table S4). This assessment uncovered 29 core bacterial families, such as *Ruminococcaceae* and *Lachnospiraceae*, found across all monkeys from every social group.

Turning to the non-core microbiome, we found overall that the difference between the mean alpha diversity for each social group fell just short of the conventional threshold for statistical significance [Shannon KW; *P* = 0.059, (Fig. S1E)]. Group F compared with social group R has significantly higher Shannon diversity. There are several bacterial families not shared among all animals in each of these groups (Table S4). The social groups F, V, and R shared the more common (pan) bacterial families, while the social groups with the most unique bacteria were groups HH and KK. Twenty-three families of bacteria were found on all members of social group HH, while 11 different bacterial families were found on all members of group KK (Table S4). These bacterial families were not universally found on all monkeys residing in the other social groups.

### Social group and beta diversity

We examined beta diversity via unweighted UniFrac distance using the complete ASV microbiome feature table, which incorporates the phylogenetic relationships of microbes between and within each group (70). We predicted that beta diversity would cluster by social group membership, and that the unweighted metric would not minimize features with very low abundance. Our results confirmed this by showing that the beta diversity by unweighted UniFrac distance matrix on a PCoA plot was significant [Adonis Pr(>F) = 0.002] by social group membership (Fig. 2D). The *R* ^2^ factor 0.07 indicated that social group membership contributed to 7% of bacterial composition variability. However, there was no significant relationship between social group and beta diversity when the weighted metric was applied to the feature table matrix (PCoA *R* ^2^ 0.084, *P* = 0.094).

### Dominance rank and alpha diversity

Next, we examined alpha diversity for the 53 monkeys with behavioral data. There was no significant association between male dominance rank and alpha diversity as measured by Shannon index. Lower-ranking females were associated with lower microbiota diversity as measured by the Shannon index compared with higher-ranking females, but not significantly (*P* = 0.09) (Fig. S2). Three outlier female samples from the same maternal line (2A5, 29Z, 52P) were previously high ranking before being displaced briefly from their social group (group F) (29). They returned to the group at a lower rank the year prior to sampling, but displayed an elevated diversity. Further investigation of social group and individuals’ maternal lines could give more insight into lower-ranking females correlating with lower microbial diversity if migrated females were removed from the analysis.

### Grooming behavior and phylogenetic alpha diversity

**Fig 3:**
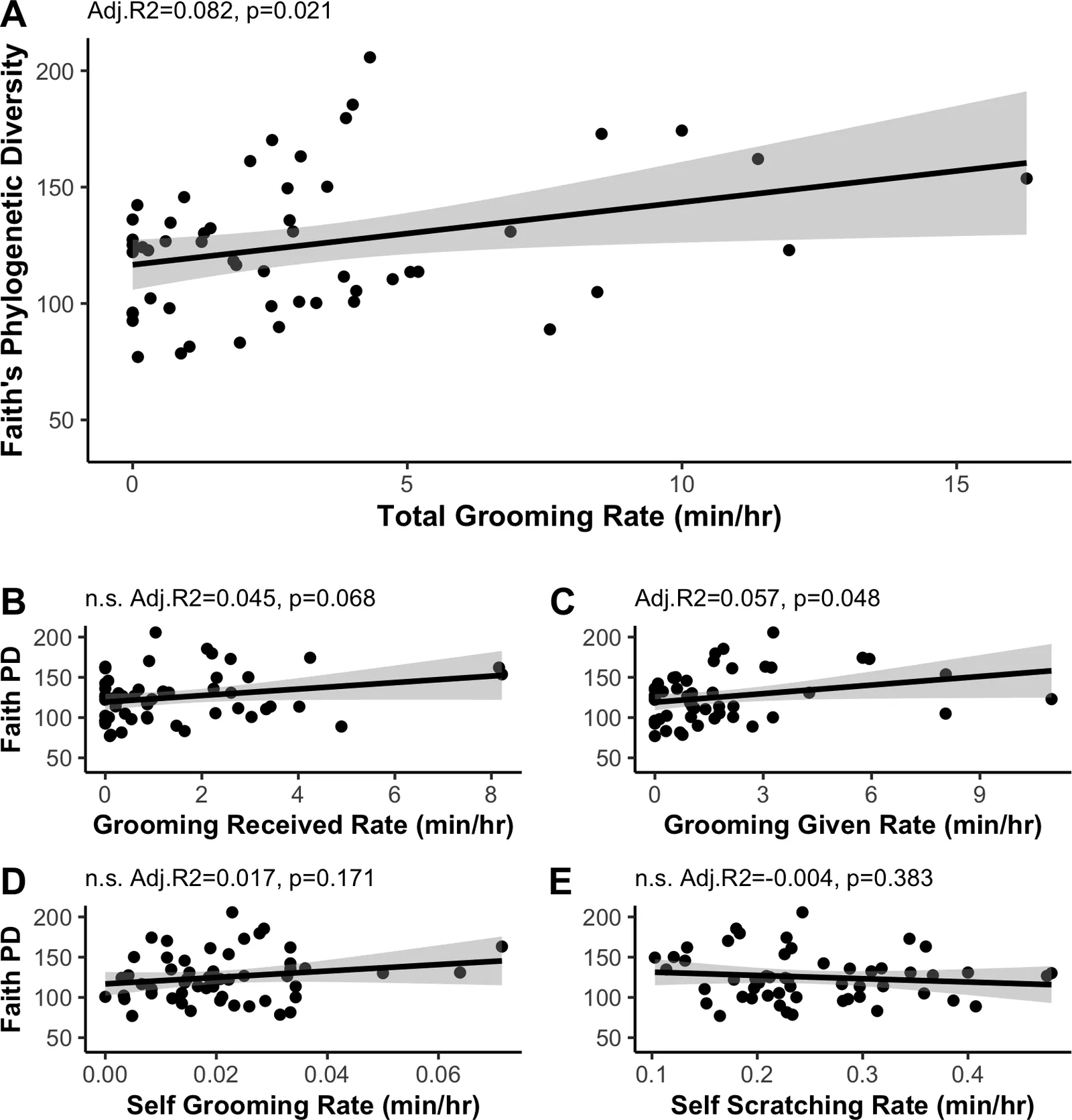
Relationships between microbiota diversity and grooming. (**A**) Scatter plot showing a significant correlation between Faith’s phylogenetic diversity measurement of alpha diversity and the rate of total time a monkey spent grooming (grooming given+ grooming received). There was a slight increase in diversity for social grooming measures for higher (**B**) rate of time receiving grooming and (**C**) rate of time giving grooming, but none for rate of time spent in self-directed behaviors, such as (**D**) self-grooming and (**E**) self-scratching.

To address our hypothesis that monkeys with higher grooming rates would display higher measures of alpha diversity, we parsed Faith’s PD from the complete microbiome feature table for a subset of monkeys with behavioral data (*n* = 53). We found a positive correlation between phylogenetic alpha diversity and the rate of total time spent engaged in social grooming (*P* = 0.02; Fig. 3A). The same alpha diversity measure was used to explore correlations between other behavioral measures, including the rate of time a monkey spent receiving grooming from another monkey and the rate of time spent giving grooming, the latter being slightly significant, indicating that a higher level of social interaction leads to more skin diversity (*P* = 0.06, *P* = 0.048, respectively; Fig. 3B and C). Compared with social behaviors, measures of SDBs (self-grooming and self-scratching; Fig. 3D and E) were not significantly associated with alpha diversity, suggesting there was a combined positive effect of total grooming (given or received) and not SDBs.

### Sampling period and skin microbe composition

**Fig 4:**
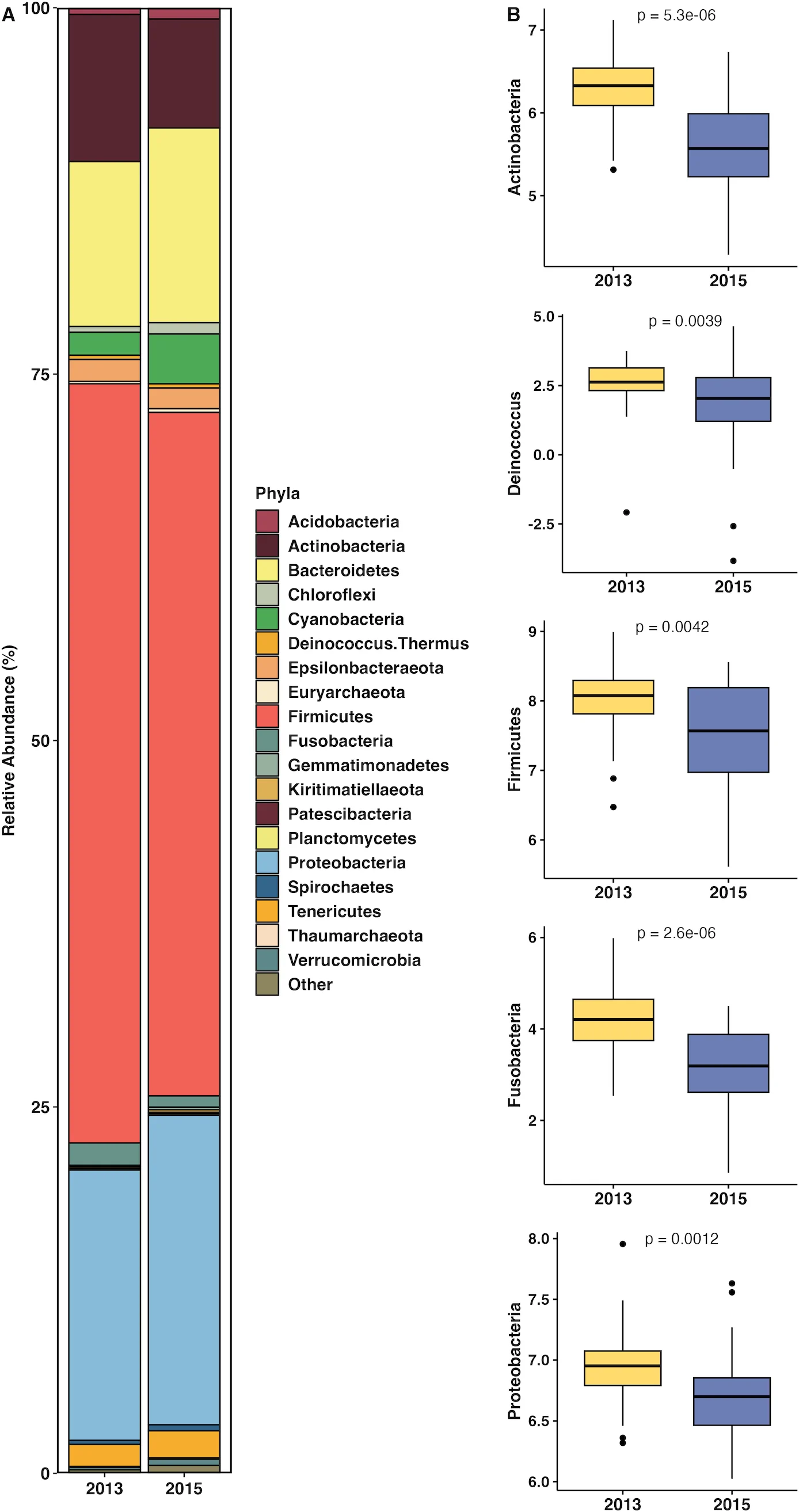
Summary of microbiome diversity according to year of sample period: 2013 (42 samples), 2015 (33 samples). (**A**) Bar plot of the average relative abundances of the top 20 total representative phyla per year. (**B**) Box plots representing the significant pairwise taxa using Wilcoxon tests on CLR transformed relative abundance values on phyla: *Actinobacteria, Firmicutes, Deinococcus-Thermus, Fusobacteria*, and *Proteobacteria*.

We assessed the relative abundance of the top 20 phyla according to the year of sampling period (2013 versus 2015) for the complete microbiome rarefied feature table (Fig. 4A). Based on the sample averages, the most common phylum, *Firmicutes*, was the most abundant of total bacteria during the 2013 sampling period (51%) compared with 2015 (46%). *Proteobacteria* was the second most prevalent phyla in the range of 18% (2013) and 21% (2015). The relative abundances for *Bacteroidetes, Actinobacteria*, and *Cyanobacteria* in 2015 were 13.3%, 7.4%, and 3.4%, respectively, and were 11.2%, 10%, and 1.6% in 2013. After CLR transformation of relative abundances, *Firmicutes*, *Actinobacteria*, and *Proteobacteria* were significantly higher in 2013 than in 2015 (Wilcoxon *P* = 0.0042, 0.0000053, 0.0012, respectively). *Deinococcus-Thermus* and *Fusobacteria* had significantly higher abundance in 2013 at 0.29% (over 2015 at 0.28%) (CLR Wilcoxon *P* = 0.0039) and 1.52% (over 2015 at 0.77%) (CLR Wilcoxon *P* = 2.6e − 06), respectively (Fig. 4B).

### Sampling period and alpha diversity

**Fig 5:**
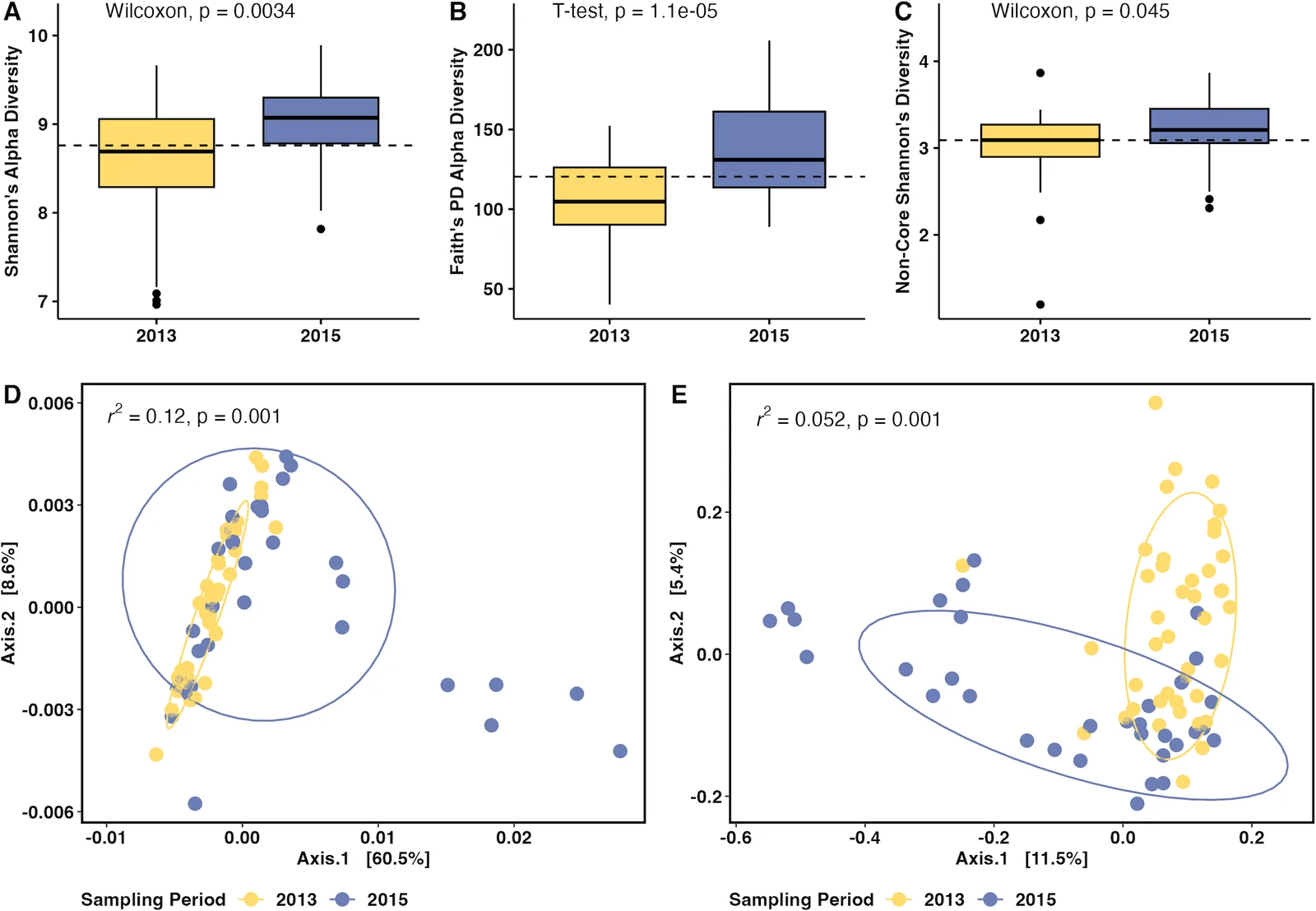
Diversity metrics according to year of sample period: 2013 and 2015. Box plots representing alpha diversity using (**A**) Shannon’s H and (**B**) Faith’s phylogenetic diversity for each monkey’s complete microbiome composition per year, as well as (**C**) Shannon’s H calculated for only the phyla level non-core taxa (complete taxa minus the core taxa found across 75 animals). (**D**) PCoA plot showing significant association between beta diversity as measured by weighted UniFrac and year of animal sampling [Adonis2 *R* ^2^ = 0.12, *P*(F-corrected) = 0.001]. (**E**) PCoA plot showing significant association between beta diversity as measured by unweighted UniFrac and year of animal sampling [Adonis2 *R* ^2^ = 0.052, *P*(F-corrected) = 0.001].

We addressed our hypothesis that environmental influences also played a role in influencing skin microbial diversity by comparing sampling periods 2013 and 2015 in the complete microbiome. Shannon’s alpha diversity differed significantly (Wilcoxon; *P* = 0.0034; Fig. 5A); if the three samples from 2014 were included as a representative third sampling period, the significance remains (Kruskal Wallis; *P* = 0.013). Faith’s PD metric of alpha diversity varied significantly by sampling period (*t*-test; *P* = 1.1 × 10^−5^), with the higher diversity measurements occurring in 2015 (Fig. 5B).

### Sampling period and core versus non-core taxonomic diversity

Tests for significant differences across sampling periods were performed using the core and non-core microbiome feature tables, both of which were parsed from the complete microbiome feature table as described above. First, we compared relative abundances of phyla represented by the core and non-core microbiome feature tables according to sampling period in bar plots (Fig. S3A and B). *Deinococcus-Thermus* was one of the most abundant phyla in the non-core microbiome and significantly higher in 2013 as stated above. Next, Shannon diversity measures for the core microbiome (at the level of phylum) fell above the conventional threshold for statistical significance across sampling periods (*P* = 0.29) (Fig. S3C). Sampling periods 2013 and 2015 shared the same richness of core microbiota diversity. When we parsed core feature tables to the level of family, we identified 29 core families of bacteria that were present on all monkeys during every sampling period (Table S5).

In contrast, Shannon diversity for the phyla level non-core microbiome was found to differ significantly across sampling periods 2013 and 2015 (Fig. 5C; *P* = 0.045). Upon finer scale, the pan and unique bacterial families are in the non-core (Table S5). For instance, *Bacteroidaceae* were present in all 2015 animals but only in 76% of 2013 animals. Collectively, these rarer microbe families were present on different monkeys during different sampling periods, suggesting they may be pervasive on the island albeit at lower abundances but higher in one or the other sampling period.

### Sampling period and beta diversity

In addition, we found support for the hypothesis that samples collected from animals within the same sampling period would be more similar than samples collected across different periods, as demonstrated by measures of weighted UniFrac beta diversity [Pr(>F) = 0.001; Fig. 5D]. Sampling period accounted for 11.8% of bacterial composition variability (Adonis2 *R* ^2^ 0.118). In addition, beta diversity by unweighted UniFrac and sampling period were also positively correlated with 5% variability [Pr(>F) = 0.001; Adonis2 *R* ^2^ 0.052], further suggesting that fluctuating environmental variation plays a role in influencing microbial composition and abundance (Fig. 5E).

### Sampling period and differentially abundant taxa

**Fig 6:**
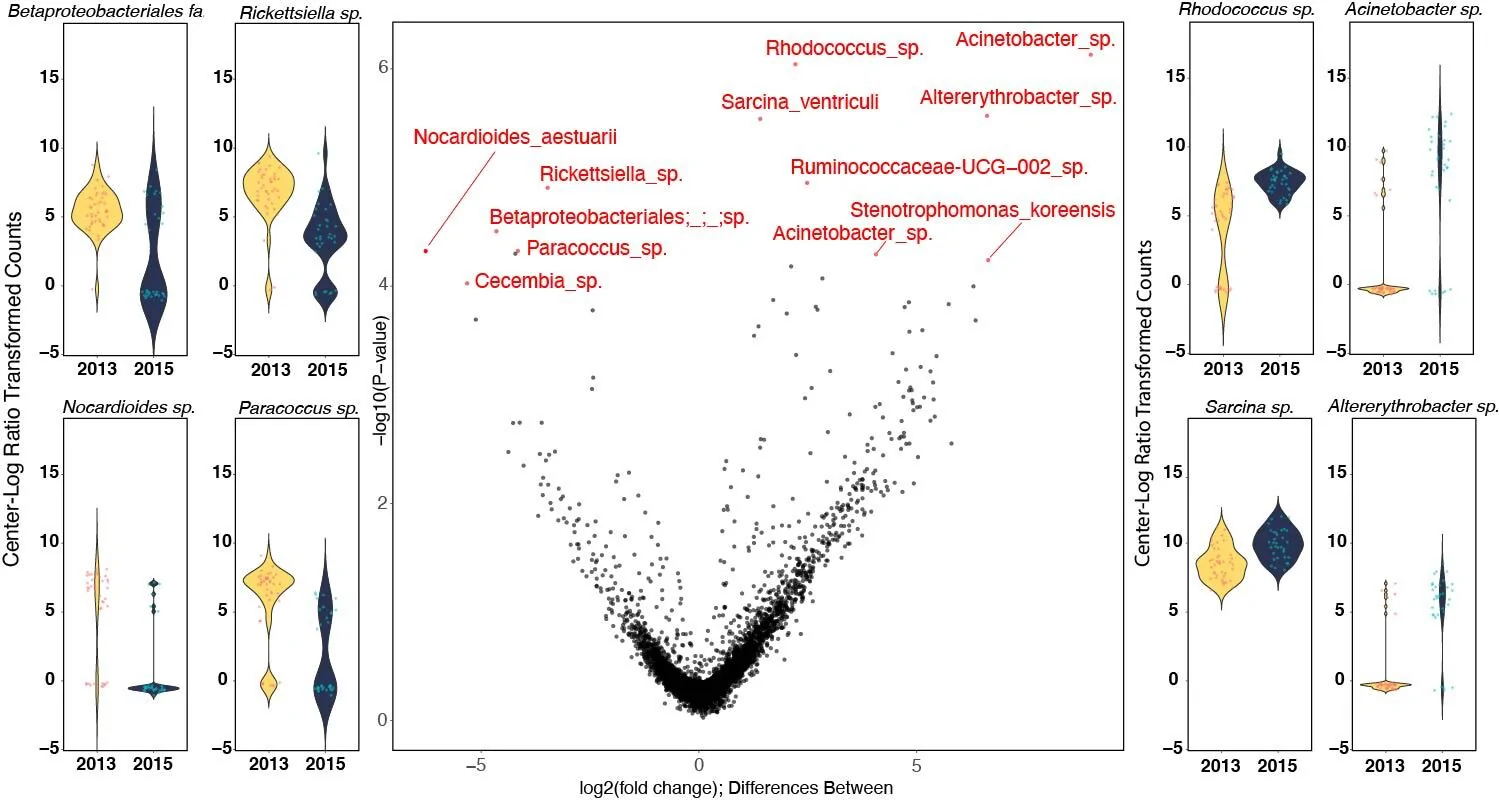
The volcano plot (center) depicts the differences between the 2013 and 2015 sampling periods for each detected ASV (red and black points). Taxa depicted in red are the most highly significant (*P* < 0.015). Overabundant ASVs from 2013 are located on the negative (left) side of the volcano plot, while overabundant ASVs from 2015 are located on the positive (right) side. The violin plots surrounding the volcano plot depict the CLR values of the most significant taxa from the volcano plot; left: overabundant taxa in 2013; right: overabundant taxa in 2015.

We compared taxa abundances from 2013 and 2015 (for *n* = 75 animals) to find differentially abundant microbial taxa. We compared these two periods because the phylogenetic difference in pairwise alpha diversity was largest between these two sampling periods (False Discovery Rate (FDR) corrected *P* = 0.0000165) and they had similar sample sizes (42 in 2013 and 33 in 2015). We therefore performed an analysis of differential abundance. The output included a total of 68 significant differentials, which included nine phyla, 13 classes, 24 orders, 41 families, 38 genera, and nine known species of bacteria and Archaea. Thirty-seven of the 68 significant features (54.4%) were found to be differentially abundant at a lower significance threshold with q-score (we.eBH *P*-value) of <0.05 (Fig. S4). When we applied our highest stringency level threshold q-score <0.015, 12 features (17.5% of 68) were the most significantly abundant taxa in one sampling period or the other as shown in the volcano plot (Fig. 6). Of these 12, seven features have higher abundance in 2015 relative to 2013. One of the most overabundant bacteria in 2013 relative to 2015 were from the phylum *Actinobacteria* (*Nocardioides*). Normalized abundance for species *Nocardioides aestuarii* is much higher for more samples in 2013 than 2015 when plotted using the CLR transformation (Fig. 6).

We performed microbe set enrichment to identify associations between differentially abundant taxa with specific diseases (list of genera searched in the MicroPattern software: *Sarcina, Acinetobacter, Rhodococcus, Rickettsiella, Stenotrophomonas, Paracoccus, Altererythrobacter, Cecembia, Ruminococcaceae, Nocardioides, Betaproteobacteriales*). The most overabundant bacterium in 2015 relative to 2013 was represented by the genus *Acinetobacter* (phylum: *Proteobacteria*). The genus *Acinetobacter* emerged as a pathway component for skin infection according to our enrichment analysis in MicroPattern (*P* = 0.0083, FDR = 0.0167); however, we were not able to distinguish the species-level identity of the 16S amplicon sequence in our data set; thus, we could not identify if it was infectious, which warrants further investigation (82). Another differentially abundant taxon, from the genus *Rhodococcus*, was also more abundant in 2015 than 2013, yet our analysis was not able to identify the species level. This genus is represented by a broad range of species, most of which are not harmful to skin.

### Sampling period and weather data

In order to address environmental differences between sampling periods, weather data were compared between the 2013 and 2015 to identify potential differences linked to variation in temperature, rainfall, and humidity. The weather during 2015 was characterized by more humid conditions (mean daily average dew point temperature: 2013 = 69.7; 2015 = 74.0; *t* = −4.55; *P* < 0.0001; mean daily average relative humidity: 2013 = 73.1; 2015 = 79.3; *t* = −3.44; *P* < 0.001). The relatively more humid conditions of 2015 were also depicted by a higher mean daily average wet bulb temperature (2013 = 73.5; 2015 = 76.2; *t* = −4.89; *P* < 0.0001), albeit with a similar daily average dry bulb temperature (2013 = 81.7; 2015 = 81.7; *t* = −0.3; *P* = 0.38).

While differences in rainfall and wind speed were not statistically significant, the weather during 2015 was characterized by higher daily measurements of rain relative to 2013 (mean daily precipitation: 2013 = 2.8 mm/day; 2015 = 3.3 mm/day; *t* = −0.48; *P* = 0.31) and by higher wind speeds (mean daily average wind speed: 2013 = 11.2 kph; 2015 = 11.9 kph; *t* = −1.24; *P* = 0.11). In order to substantiate consistent atmospheric differences between 2013 and 2015, we expanded the analysis to include the preceding month of weather data for each sampling period. When these data from September 2013 and September 2015 were included in our comparisons, we found consistent and concordant statistical differences in mean daily precipitation and mean daily average wind speed by sampling period (Table S6).

## DISCUSSION

Our analysis of 78 skin swabs indicated that yearly environmental factors strongly influence the diversity and composition of the skin microbiota of rhesus macaques living on Cayo Santiago, Puerto Rico. Social group membership and social behavioral interactions between monkeys also account for some of the variation, albeit with less influence. Our work supports previous research indicating a higher diversity of skin microbiota on non-human primate skin than on humans (7, 26). Overall, our characterization of the skin microbiome of macaques is consistent with previous studies of human skin indicating the most abundant phyla comprising *Actinobacteria*, *Firmicutes*, *Bacteroidetes,* and *Proteobacteria* (83). The top three most abundant phyla (*Firmicutes, Bacteroidetes,* and *Proteobacteria*) were also reported in oral, vaginal, and anal swabbed samples from macaques (78). While the skin of other mammals comprises higher proportional abundances of *Bacteroidetes* taxa relative to taxa within the phylum *Actinobacteria*, our results suggest that the skin of rhesus macaques harbors closer proportional abundances (84). In addition, *Chloroflexi* was found in higher proportions relative to *Actinobacteria* on the skin of other mammals, but we found the opposite on the skin of rhesus macaques on Cayo Santiago (84).

### Sex, rank, and age are weakly associated with skin microbiome diversity

We did not find a strong relationship between variation in microbial alpha diversity and dominance rank or overall age among rhesus macaques. Yet, when we examined these factors according to sex, we found tentative support for sex-specific impacts of age. Among males, skin microbiome diversity was reduced in older monkeys, whereas females did not show this association. Dominance rank potentially predicted skin microbial diversity for females, but not males. Apart from a few outlier low-ranking female monkeys that had higher ranks before temporarily moving from group F to small social sub-groups (OO and NN), female rhesus macaques with lower dominance ranks had lower measures of skin microbiome diversity. Unless social groups fission, which occurs seldomly, then females typically remain in their social group throughout their life while inheriting their rank along maternal lines (31), suggesting high-ranking matrilines coupled with patterns of female philopatry have enhanced skin microbe diversity. Additional research that broadens the sample size and frequencies of samples from older adult males and females will be required to explore the effects of age and ranks in greater detail.

### Social group and grooming behavior influences skin microbial composition

Tung et al. showed that baboons in the same social group demonstrated more similar gut microbiome composition (11). We hypothesized that rhesus macaques would display variation in microbial diversity in accordance with social group membership and behavioral metrics (i.e., grooming, SDBs), yet these hypotheses were met with mixed results. While no significant associations were found between social group membership and the complete assemblage of axillary skin microbiome features as measured by alpha diversity, parsed assemblages representing the core and non-core phyla were variable in both evenness and richness across social groups. Total compositional taxonomic diversity revealed that skin microbiomes on rhesus macaques were more phylogenetically similar as measured within social group members rather than between social groups, which is consistent with a previous study of gut microbes in endangered ring-tailed lemurs in southwestern Madagascar (9). There, researchers found no significant differences in gut microbial alpha diversity across social groups, and this result was consistent with frequent migration patterns and overlapping home ranges. In the population of macaques in our study, adult males tend to disperse from their natal social groups to join new groups, and this dispersal, combined with significant overlap of territories for some of the social groups, may help explain the lack of clear differences in alpha diversity across social groups. At the same time, we found a significant difference between the core microbiome on monkeys in social group F compared with monkeys in social group V, suggesting social groups could be characterized by the more common taxa of bacteria, but not so with the least ubiquitous taxa, or those which comprised the non-core microbiome. Variability between these two social groups could be influenced by several factors, including changes in the environment between sampling period years. Sampling effort was balanced across years for each of these two social groups (group F: 2013: 23, 2014: 0, 2015: 20; group V: 2013: 7, 2014: 1, 2015: 4).

We found several weak associations that should be interpreted with caution due to the nature of our sampling. Some social groups, for instance, were represented by less than 10 animals, and their axillary samples were collected across multiple trap months and years (2, 3, 11). In addition, social group F was the largest social group and encompassed a greater area of the island compared with other social groups, adding to the complexity of social group dynamics. Group V spends more time on the sub-island of Cayo than any other group contributing to spatial factors and possibly influencing this group’s lower microbial diversity. Finally, the large number of low abundant rare features greatly contributed to the total skin microbiota, and were minimized when weight (quantitative measure) was added to distance metrics in beta diversity. This factor potentially diminished the phylogenetic taxonomic composition compared between several social groups. Additionally, the rare phyla in the non-core microbiota did not have a large impact on the variability between social groups.

Earlier studies of this population demonstrated how social interactions, defined by grooming behavior, were consistent for group F females until nearing time to eviction from their social network, at which time they became more discriminating and groomed with close kin (29, 31, 33). Females having a strong grooming connection to a top partner gives that female a higher chance of survival (29, 31, 33). Our assessment revealed a positive association between social grooming behavior and phylogenetic diversity of skin microbes, and this result corroborates an earlier finding from a captive population of rhesus macaques whereby grooming and huddling tendencies were linked to more similar gut microbiomes (20). We speculate that social grooming interactions gave rhesus macaques more opportunities to share phylogenetically similar skin microbes compared to nonsocial physical interactions (like SDBs).

### Yearly differences influence skin microbiota

We hypothesized that skin microbes on rhesus macaques would exhibit yearly differences based on the date they were collected, and in fact, the most salient signals for microbial diversity were in accordance with the different sampling periods. Similar effects were observed using microbiome data generated from skin samples collected from dogs, whereby diversity across seasonally collected samples was significantly higher than among those collected within the same season (23), indicating that time and environment can influence microbial diversity.

We found microbial differences in phylogenetic diversity and abundance in 2013 versus 2015. The same effect was true regarding richness and evenness of features, corroborating a seasonal effect found in the gut mycobiomes of Tibetan macaques (19). The non-core microbiome taxa in rhesus macaque skin samples illustrate the variability of abundance and evenness of rare phyla that fluctuate between sampling periods. The total skin microbiota is visually distinguishable between 2013 and 2015. In addition, when the distance metric was weighted in beta diversity, rare taxa were not overshadowed. We identified differentially abundant bacteria between 2013 and 2015, which represented the most phylogenetically diverse sampling periods with relatively similar sampling sizes. For example, the highly abundant 2013 bacterium *Nocardioides aestuarii* was first isolated from a Korean tidal flat sediment (85) bacterium and may be differentially abundant between sampling periods due to the drier air in 2013. Another highly abundant 2013 bacterium, *Rickettsiella,* is found within the cells of arthropods, which are commonly found on macaque fur. With a less humid climate in 2013, arthropods, such as ticks, may have thrived and transmitted *Rickettsiella* to the fur and skin of rhesus macaques. The bacterium that is the most abundant during both 2013 and 2015, but with a slightly higher level in 2015, is the *Firmicute Sarcina ventriculi*. With anaerobic metabolism, it can often be found in stomachs of mammals, but also in soil or mud.

Differentially abundant bacteria may have diverged due to differences in humidity levels. These types of environmental effects were found in other primates. For example, the cyclical wet and dry seasons in Costa Rica influenced the gut microbiomes of Capuchins, and the differentially abundant skin taxa of rhesus macaque that we identified in this study spanned similar phyla (*Firmicutes* and *Proteobacteria*) (21). However, we also found differentially abundant taxa in the phylum *Actinobacteria*. Further monitoring is necessary, especially with regard to functional impact, since the genus *Acinetobacter* was significantly abundant on macaque skin during the more humid 2015 season, whereas the same genus was found in the gut of Capuchins during the late dry season (21).

We found the alpha diversity between the two largest social groups with the most diverse microbiota, F and V, to not be significantly different and did not pursue additional tests to explore social group pairwise differential abundance tests. Because we controlled for sampling period in our batch correction method, we cannot surmise if social group or behavior exerts further influences on the effect of microbe seasonality. We recognize that strong and salient signals from yearly impacted environmental factors may have overshadowed any potential correlations between microbial diversity and social behaviors. In the future, within-year sampling of skin microbes in social groups that have larger sample sizes will help to uncover potential finer-grain differences associated with social behaviors, much in the same way that gut microbiome research has exposed the interplay between the environment and social behaviors that contribute to the overall diversity of bacteria in the gut (17 – 21, 86). Further studies could include the maternal line data to assess if the skin microbiota is heritable in social groups and associated with age or environment, similar to the gut microbiome in wild baboons (76). The results of our study allude to the potential importance of the broader environment in modulating the skin microbiome, and by extension, the protective abilities of our body’s first line of defense. Understanding how the environment and social network impact primate skin microbiota will help determine how we can manipulate the skin microbiota for improved health in humans and research animals in the future.

### Conclusions

This study has uncovered associations between microbe diversity and social group membership and, to a larger extent, sampling periods. We found a core set of phyla on the skin of every sampled rhesus macaque from all social groups, but the most common core phyla varied in their abundance according to social group membership. We also found that the full diversity of skin microbes, as well as rare non-core phyla, varied significantly by year of sampling period. In addition, rhesus macaques that spent more time social grooming were characterized by more diverse skin microbes. Several variables, such as the overlap of territories, can influence these weak associations between the microbial diversity and social behavior, and greater sample sizes are needed for some groups to validate with increased statistical power. We hypothesized that social group membership would show a positive correlation with skin microbes, as previously shown for gut microbes in different non-human primate populations. Yet after assessments of group dynamics and social behaviors, we recognize that larger sample sizes correlating to a single sampling period are likely required to potentially uncover effects that are not masked due to stronger yearly environmental factors. While our results uncovered correlations, further research is necessary to determine the functional and health-related impacts of environmental influences on skin microbial diversity.

Our study demonstrates the influence of year-to-year environment on skin microbes in the axillary region of macaques on Cayo Santiago, Puerto Rico. We set the stage for continued skin microbiome research in anticipation that this trajectory, combined with our other behavioral, genetic, and health data in the same monkeys, will potentially shed light on the connections between behavior, microbiome, and interactions with the external environment.

## Data Availability

The raw sequence data for all samples were deposited as fastq-formatted files in NCBI’s Sequence Read Archive (SRA) repository (BioProject PRJNA675026; accession numbers in Table S2).

## AUTHOR NOTE

All data analysis scripts can be found here:

https://github.com/CayoBiobankResearchUnit/macaque-skin-microbiome.git

## SUPPLEMENTAL MATERIAL

Supplemental material spectrum.02974-23-s0001.pdf Supplemental figures.

Supplemental file 1 spectrum.02974-23-s0002.xlsx Table S1.

Supplemental file 2 spectrum.02974-23-s0003.xlsx Table S2.

Supplemental file 3 spectrum.02974-23-s0004.csv Table S3

Supplemental file 4 spectrum.02974-23-s0005.xlsx Table S4.

Supplemental file 5 spectrum.02974-23-s0006.xlsx Table S5.

Supplemental file 6 spectrum.02974-23-s0007.xlsx Table S6.

ASM does not own the copyrights to Supplemental Material that may be linked to, or accessed through, an article. The authors have granted ASM a non-exclusive, world-wide license to publish the Supplemental Material files. Please contact the corresponding author directly for reuse.
