# Supplementary material for: Yearly variation coupled with social interactions shape the skin microbiome in free-ranging rhesus macaques
Source: Microbiol Spectr. 2023 Sep 26;11(5):e02974-23. doi: 10.1128/spectrum.02974-23 (PMC10580906; doi:10.1128/spectrum.02974-23)
Supplement: Supplemental material — Supplemental figures. [file spectrum.02974-23-s0001.pdf]

Supplemental Figures

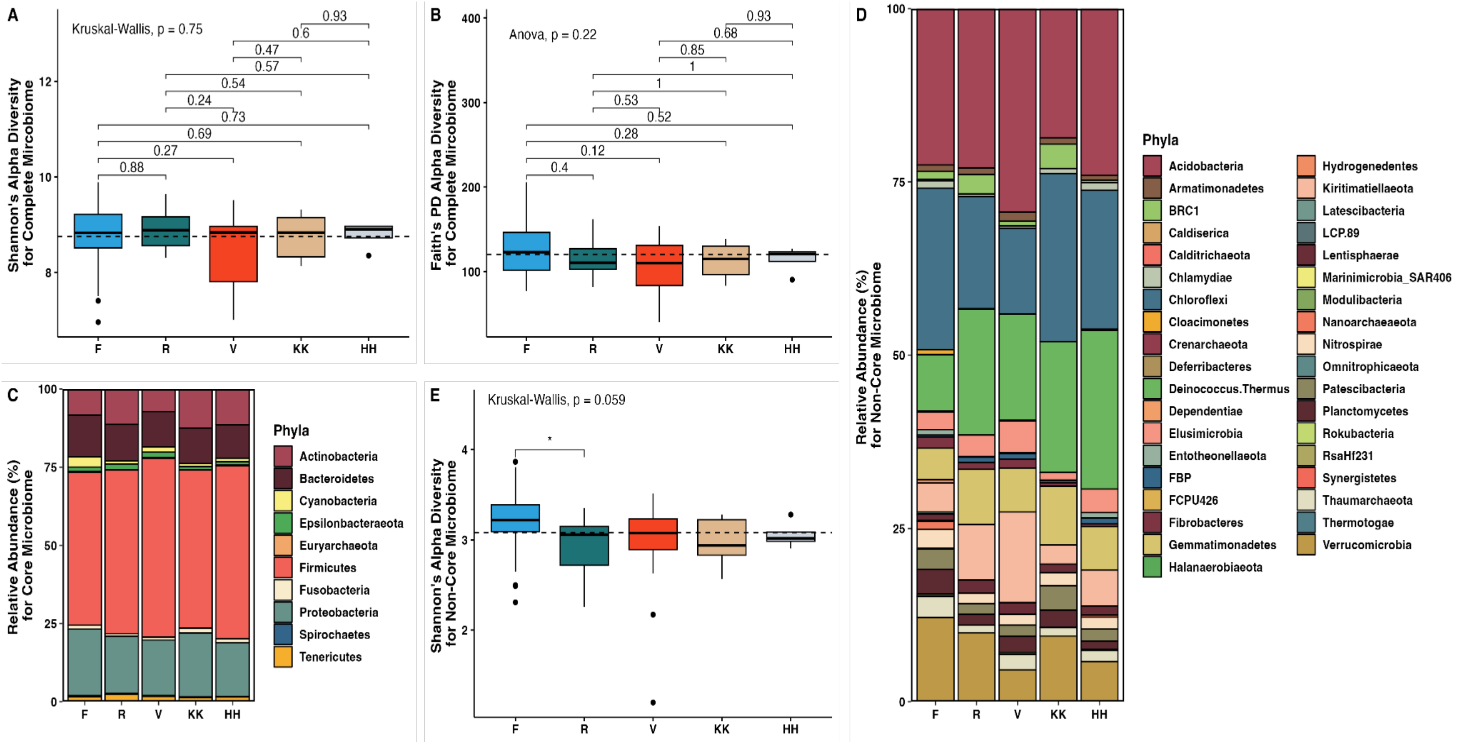

Figure S1: Complete microbiome alpha diversity according to social group using (A) Shannon and (B) Faith's phylogenetic metrics; (C) average relative abundance of all core and (D) non-core phyla in bar plots across social groups; (E) non-core microbiome Shannon diversity for social group.

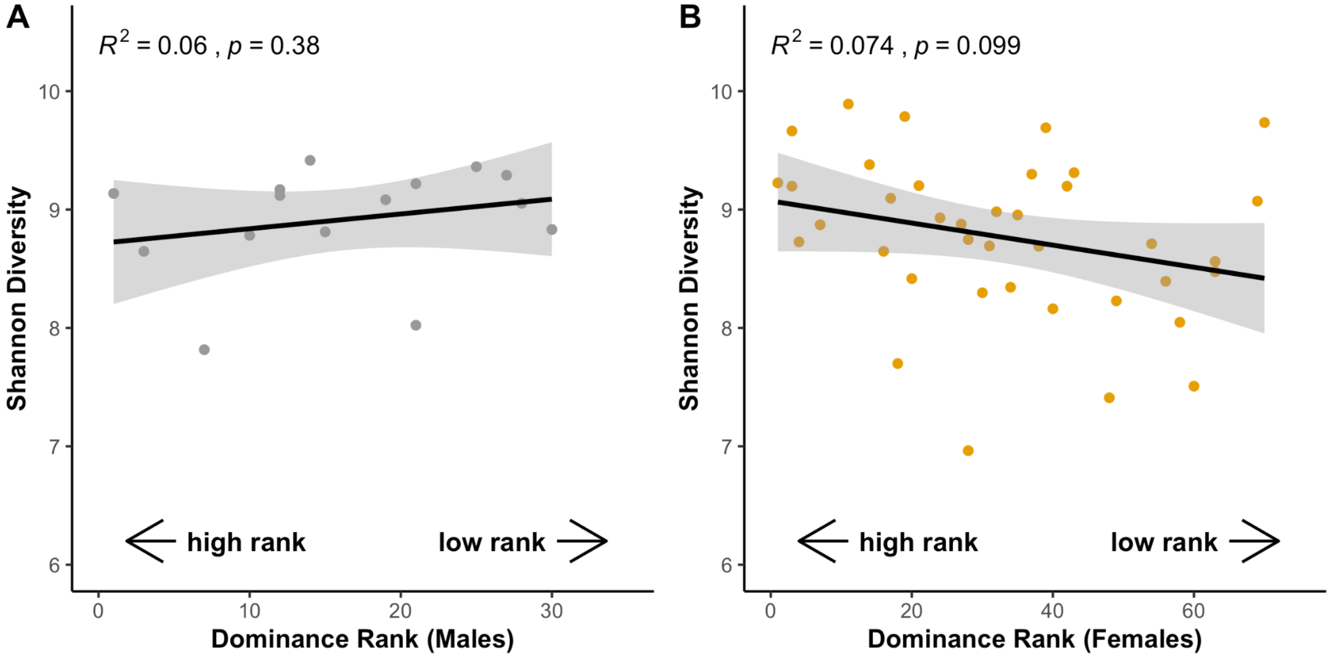

Figure S2. Scatter plots of Shannon alpha diversity according to dominance ordinal rank for (A) males and (B) females. Lower ordinal rank numbers represent animals with higher rank within their social group.

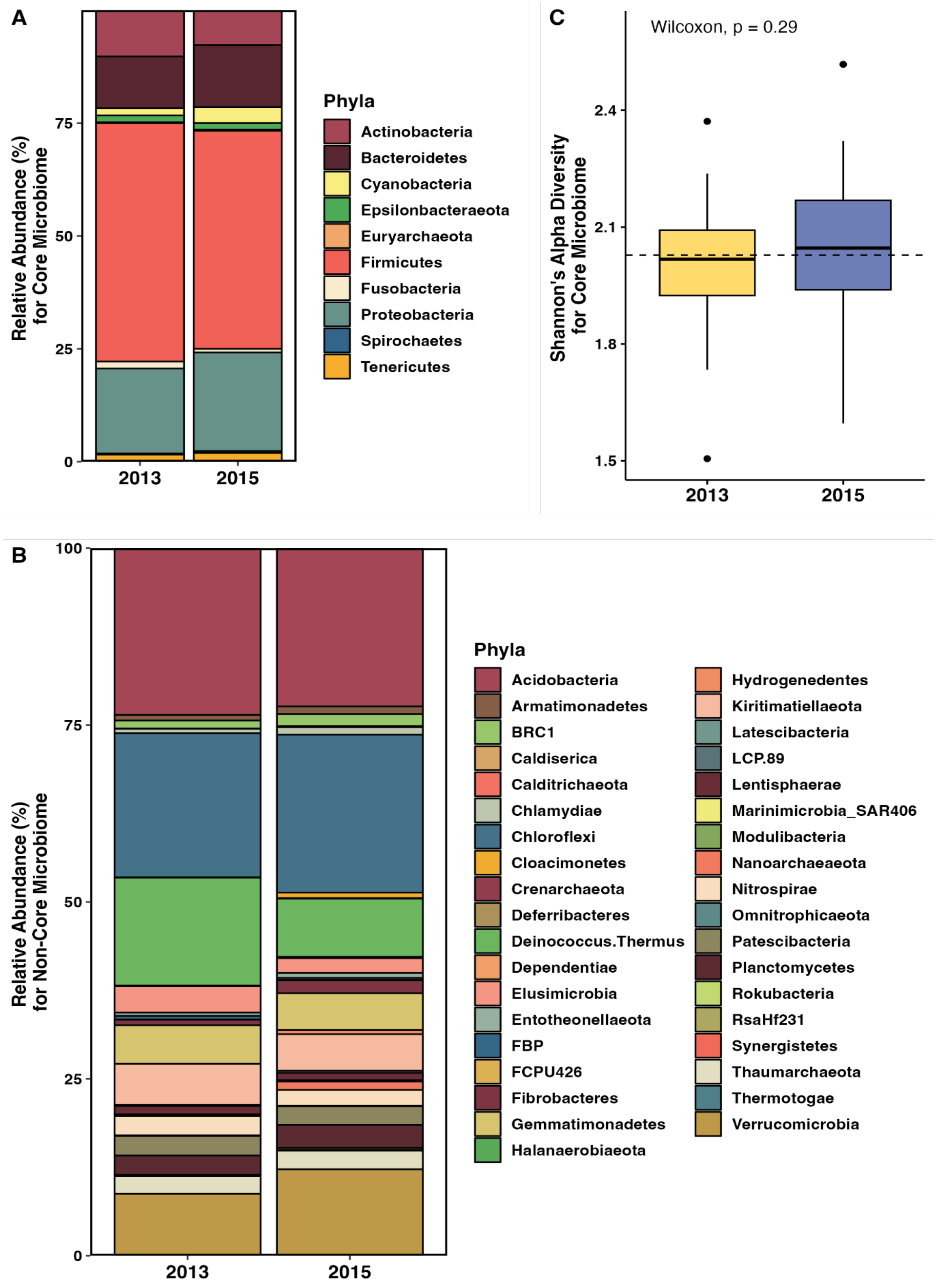

Figure S3: (A) Average relative abundance of core phyla and (B) non-core phyla in bar plots for Sampling Period. (C) Core microbiome Shannon box plot using Wilcoxon test.

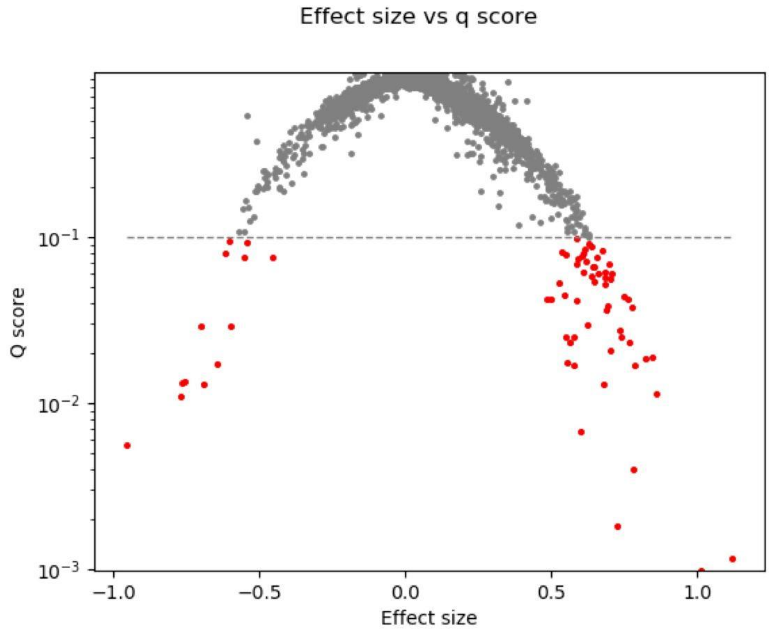

Figure S4. Plot displaying the effect size versus q-score (<0.1) for differential abundant features in microbiomes according to Sampling Period. Red points represent features with statistical significance while gray dots represent features with no statistical significance. Overabundant features in samples collected in 2013 are found on the left side of the plot, while overabundant features in samples collected in 2015 are on the right side of the plot.
